# Loss of IKKβ but Not NF-κB p65 Skews Differentiation towards Myeloid over Erythroid Commitment and Increases Myeloid Progenitor Self-Renewal and Functional Long-Term Hematopoietic Stem Cells

**DOI:** 10.1371/journal.pone.0130441

**Published:** 2015-06-23

**Authors:** Jing Zhang, Li Li, Albert S. Baldwin, Alan D. Friedman, Ido Paz-Priel

**Affiliations:** 1 Division of Pediatric Oncology, Johns Hopkins University School of Medicine, Baltimore, Maryland, United States of America; 2 Lineberger Comprehensive Cancer Center and Department of Biology, University of North Carolina, Chapel Hill, North Carolina, United States of America; Johns Hopkins School of Medicine, UNITED STATES

## Abstract

NF-κB is an important regulator of both differentiation and function of lineage-committed hematopoietic cells. Targeted deletion of IκB kinase (IKK) β results in altered cytokine signaling and marked neutrophilia. To investigate the role of IKKβ in regulation of hematopoiesis, we employed Mx1-Cre mediated IKKβ conditional knockout mice. As previously reported, deletion of IKKβ in hematopoietic cells results in neutrophilia, and we now also noted decreased monocytes and modest anemia. Granulocyte-macrophage progenitors (GMPs) accumulated markedly in bone marrow of IKKβ deleted mice whereas the proportion and number of megakaryocyte-erythrocyte progenitors (MEP) decreased. Accordingly, we found a significantly reduced frequency of proerythroblasts and basophilic and polychromatic erythroblasts, and IKKβ-deficient bone marrow cells yielded a significantly decreased number of BFU-E compared to wild type. These changes are associated with elevated expression of C/EBPα, Gfi1, and PU.1 and diminished Gata1, Klf1, and SCL/Tal1 in IKKβ deficient Lineage^-^Sca1^+^c-Kit^+^ (LSK) cells. In contrast, no effect on erythropoiesis or expression of lineage-related transcription factors was found in marrow lacking NF-κB p65. Bone marrow from IKKβ knockout mice has elevated numbers of phenotypic long and short term hematopoietic stem cells (HSC). A similar increase was observed when IKKβ was deleted after marrow transplantation into a wild type host, indicating cell autonomous expansion. Myeloid progenitors from IKKβ- but not p65-deleted mice demonstrate increased serial replating in colony-forming assays, indicating increased cell autonomous self-renewal capacity. In addition, in a competitive repopulation assay deletion of IKKβ resulted in a stable advantage of bone marrow derived from IKKβ knockout mice. In summary, loss of IKKβ resulted in significant effects on hematopoiesis not seen upon NF-κB p65 deletion. These include increased myeloid and reduced erythroid transcription factors, skewing differentiation towards myeloid over erythroid differentiation, increased progenitor self-renewal, and increased number of functional long term HSCs. These data inform ongoing efforts to develop IKK inhibitors for clinical use.

## Introduction

Hematopoiesis is a tightly regulated process in which pluripotent hematopoietic stem cells (HSC) differentiate into lineage specific progenitors and mature subsequently into specialized hematopoietic cells [1]. HSCs maintain a balance between self-renewal and differentiation to support hematopoiesis throughout the lifetime of the organism.

NF-κB is a family of closely related dimeric transcription factors [2]. Five members are recognized in mammalian cells: RelA/p65, Rel B, c-Rel, NF-κB1/p50 and NF-κB2/p52; all sharing a Rel homology domain that mediates dimerization and DNA binding. The most prevalent species is the NF-κB p50:p65 heterodimer that is sequestered in the cytoplasm bound to a member of the IB family of inhibitors (IBα, IBβ, or IBε) or the precursor proteins p100 or p105. Upon stimulation, IB is phosphorylated by IB kinase (IKK), ubiquitinated, and undergoes proteasomal degradation to allow active dimers to translocate into the nucleus and bind target DNA κB sites. The IKK complex is comprised of two catalytic subunits IKKα and IKKβ, and a regulatory subunit IKKγ (or NEMO). While IKKα regulates non-canonical NF-κB signaling, IKKβ participates in the regulation of the canonical pathway, which typically culminates in translocation of p50:p65 dimers to the nucleus. In addition to phosphorylating IB proteins, other IKK substrates modulate immune response, chromatin remodeling, and autophagy [3–8].

Through various mechanisms, constitutive activation of NF-B is encountered in multiple human malignancies [9–11]. Moreover, accumulating evidence points to the importance of this pathway in cancer initiating cells of different origin such as muscle, breast, prostate, or bone marrow [12–15]. Thus, NF-B is a potentially attractive therapeutic target for both inflammatory and malignant conditions [9,16]. NF-κB is activated in cells expressing BCR-ABL1, and inhibition of IKK compromises leukemogenesis and enhances the sensitivity of cells to imatinib or dasatinib [17]. Guzman et al. reported activation of NF-B in human AML samples and in the early CD34^+^ population and a differential sensitivity to NF-κB inhibition between normal and leukemic stem cells [12,18,19]. Recently, constitutive activation of NF-κB by autocrine secretion of TNFα was shown to be essential for myeloid leukemia progression and leukemia initiating cells expansion [15]. Efforts are underway to target this pathway pharmacologically via proteasome or IKK inhibition in the clinical or preclinical stages. However, much less is known about the role of NF-κB in normal hematopoiesis. Concerns may arise regarding the safety of prolonged exposure to IKKβ inhibitors and its effects on the normal hematopoietic stem and progenitor cells. Further understanding of the effects of p65 or IKKβ loss on early hematopoietic progenitors and stem cells is required to inform efforts to inhibit this pathway with small molecules in the clinic.

Loss of either RelA or IKKβ is embryonic lethal due to TNFα-induced massive apoptosis of hepatocytes [20,21], highlighting the central role of IKKβ in canonical NF-κB activation. Therefore, mouse models relying on conditional or tissue specific deletion of RelA or IKKβ were developed [22–27]. Deletion of IKKβ in hematopoietic cells using Mx1-Cre results in increased granulocyte/monocyte progenitors (GMP) and marked peripheral neutrophilia [22,28] secondary to release of IL-1β primarily from myeloid cells [25,28]. IL-1β induces increased IL-17 production and expansion of CD4^+^ Th17 cells which in turn excrete G-CSF to promote expansion of GMPs [25,28]. Germline mutations of the *IKBKB* gene were reported in patients who suffer from severe combined immunodeficiency [29,30]. Targeted loss of NF-B p65/RelA in the hematopoietic compartment using Vav-Cre results in cell autonomous accumulation of long and short term HSCs but a decrease of committed progenitors including common myeloid progenitor (CMP), granulocyte/monocyte progenitor (GMP), megakaryocyte/erythroid progenitor (MEP), and common lymphoid progenitor (CLP) fractions [26]. Of note, HSC lacking p65 had a disadvantage in the competitive repopulation assay [26].

Although IKKβ and p65 are intimately related in the canonical NF-κB pathway, the effects of targeting these proteins using a similar experimental system were never directly evaluated. Here we show by comparing marrow lacking IKKβ or p65 due to Mx1-Cre induction, that loss of IKKβ but not p65 is associated with increased myeloid progenitor replating capacity, and increased number of functional long term HSCs (LT-HSC) as assessed by competitive repopulation. While p65 deletion is associated with a modest myeloid expansion and normal erythropoiesis, IKKβ deficient hematopoiesis is characterized by increased GMPs and reduced MEPs reflecting a skewed lineage commitment favoring myeloid over erythroid fate. This shift may be explained in part by diminished Gata1 and increased PU.1 and C/EBPα expression specifically in Lineage^-^Sca1^+^c-Kit^+^ (LSK) cells lacking IKKβ. Together these data indicate a critical role for IKKβ in HSC homeostasis that is only in part NF-κB dependent.

## Materials and Methods

### Mice

Mice harboring floxed *Ikk*β or floxed *RelA* (p65) alleles were previously described [22,24,27,31]. These were bred with Mx1-Cre mice (Jackson Laboratories) to generate C57BL/6 IKKβ(f/f);Mx1-Cre or p65(f/f);Mx1-Cre mice. IKKβ(f/f) or p65(f/f) littermates were employed as control. 6–8 week-old mice were injected with 400 μg copolymer of polyinosinic and polycytidylic acids [poly(I:C)] intraperitoneally every other day for 7 injections, and bone marrow or spleen cells were collected 6 weeks after the first injection. We allowed this rest period as poly(I:C) may influence proliferation, survival, and differentiation [32,33]. Single cell suspensions from bone marrow or spleen cells were obtained using a 40 μm cell strainer, and red cells were lysed with NH_4_Cl buffer.

In transplantation experiments, congenic C57BL/6 CD45.1^+^ recipient mice were lethally irradiated using a single dose of 950 cGy, delivered 4 hrs prior to tail vein injection with 1E6 CD45.2 marrow cells from wild type, IKKβ(f/f);Mx1-Cre, or p65(f/f);Mx1-Cre donor mice which were not previously exposed to poly(I:C). Eight weeks after transplantation mice were injected with poly(I:C) as described above and bone marrow cells were harvested and analyzed 6 wks later.

For competitive repopulation assay congenic C57BL/6 CD45.1^+^ recipient mice were irradiated as described above, and received a total of 5E5 IKKβ^Δ/Δ^ CD45.2 and 5E5 CD45.1 wild type unsorted marrow cells at a 1:1 IKKβ^Δ/Δ^: wild type ratio. Using anti-CD45.1 and anti-CD45.2 we employed flow cytometry to analyze their relative contributions to the marrow at 20 wks post-transplantation.

For complete blood count analysis mice were bled approximately 25 μL by submandibular bleeding [34] and analyzed immediately using the Hemavet950 system (Drew Scientific).

### Ethics statement

All animal experiments were performed in strict accordance with the recommendations in the Guide for the Care and Use of Laboratory Animals of the National Institutes of Health. The protocol was approved by the Johns Hopkins University Institutional Animal Care and Use Committee. All efforts were made to limit the number of animals used to minimize suffering and discomfort.

### FACS analysis and flow cytometry

Single cell suspensions of bone marrow cells were washed with phosphate-buffered saline (PBS), 1% heat inactivated fetal bovine serum (HI-FBS) and incubated on ice with the following anti-mouse monoclonal antibodies and dyes: PerCP Cy5.5-anti-Mac-1 (M1/70), FITC- or allophycocyanin (APC)–anti-Gr-1, PerCP-Cy5.5–anti-Sca-1 (D7), FITC-anti-CD34 (RAM34), PerCP-Cy5.5-anti-B220 (RA3-6B2), APC-anti-CD71 (R17217), and PerCP Cy5.5-streptavidin (eBioscience, San Diego, CA); APC—anti-c-kit (2B8), PE-anti-CD135 (A2F10.1), PE—anti-FcγIII/IIR or CD16/32 (2.4G2), PE-anti-Ter119 (Ter119), FITC-anti-CD45.1 (A20), FITC-Streptavidin, and biotin-conjugated mouse Lineage Cocktail (BD Pharmingen, San Jose, CA); APC-anti-CD45.2 (104), FITC-anti-Gr-1 (RB6-8C5), biotin-conjugated CD115/CSF-1R (AFS98), and APC-Streptavidin (BioLegend, San Diego, CA); PE—anti-F4/80 (Caltag, Burlingame, CA); or Alexa Fluor 488-anti-Annexin V (Life Technologies, Grand Island, NY). Flow cytometry analysis was performed using a BD FACSCalibur machine (BD Biosciences), and data were interpreted using FloJo Cytometric Analytical software (TreeStar).

For DNA content analysis cells were sorted based on surface markers, permeabilized in PBS with 0.05% Tween-20 and denatured in 2 M HCl with 0.2 mg/ml pepsin. Cell pellets were neutralized with borate buffer (boric acid 100 mM, NaCl 75 mM, and sodium tetraborate 25 mM) and washed with PBS. RNA was degraded by treatment with RNase at 37°C for 30 min, and DNA was labeled with propidium iodide (25 μg/mL). Cellular DNA content was then measured using a BD Facscaliber flow cytometer. Sub-cellular debris was gated out and singlet discrimination was performed by gating on FL2-A and FL2-W channels. Cell cycle was estimated using FloJo Cytometric Analytical software.

### Progenitor assays

For myeloid colonies, 1E4 total bone marrow cells were plated in 1 mL methylcellulose medium (Methocult M3231, Stem Cell Technologies, Vancouver, BC, Canada) with IMDM, 10% HI-FBS supplemented with 10 ng/mL murine IL-3, 10 ng/mL murine IL-6, and 50 ng/mL murine SCF. Colonies of at least 50 cells were counted between days 7 and 8. BFU-E were enumerated 8 days after 2E5 marrow cells per 1 mL were cultured in 40% MethoCult M3120, IMDM, 10% plasma-derived serum, 20% BIT (Stem Cell Technologies), 5% protein free hybridoma medium (PFHM), 2 mM glutamine, 55 nM β-mercaptoethanol, and 10 U/mL hEPO. For the serial replating assay, total colony cells were washed in sterile PBS and 1E4 cells in 1 mL methylcellulose medium were replated every 7 days for up to 6 rounds. A minimum of three independent experiments were performed in triplicates for each of the colony assays.

### Quantitative reverse-transcription PCR and Western blotting

Total RNA was isolated and first strand cDNA was synthesized and assayed in triplicate as previously described [11]. Amplification of the endogenous murine large ribosomal subunit (mS16) transcript was used as a reference to standardize between samples and fold expression was calculated as described [11]. Each experiment was repeated at least three times. Oligonucleotides employed were custom ordered from Sigma-Aldrich, and their sequences are presented in Table 1.

**Table 1:** Primers used for Real-time PCR analysis.

Gene	Forward sequence	Reverse sequence
**Real time PCR**		
Axin2	CGGCTGCGCTTTGATAAGG	GTGAGCCTCCTCTCTTTTACAGC
Bcl2	CATACATTATAAGCTGTCACAG	GTTGCTCTCAGGCTGGGAAG
Bcl-w	CAGTGAGGACAGTGCTGAC	CTATTGTTCCAGCTCTCCTG
Bcl-xL	AACTCTTTCGGGATGGAGTAAA	GTGGTCATTCAGATAGGTGGC
C/EBPα	CGGTGGACAAGAACAGCAAC	CGGAATCTCCTAGTCCTGGC
C/EBPβ	GTTTCGGGACTTGATGCAAT	CCCCGCAGGAACATCTTTA
C/EBPε	AGTACCAAGTGGCACACTGC	GAGAAGGGGACTGCAGGGA
Gata1	CAGAACCGGCCTCTCATC	TCCGCCAGAGTGTTGTAGTG
Gata2	ACGCCTGTGGCCTCTACTAC	GGATTTGCTGGACATCTTCC
Gfi1	TCCCTGTCAGTACTGTGGCA	TGGAGCTCTGACTGAAGGCT
HoxA9	ACAATGCCGAGAATGAGAGC	CGCTTCTTCCGAGTGGAG
IKKβ	AAGAACAGAGACCGCTGGTG	TCCTTGCTGCAGAACGATGT
Irf8	AGCAGGATTACAATCAGGAGGT	TCGGGGACAATTCGGTAAACT
Klf1	CTTTGGCACCTAAGAGGCAG	CAGGAGCAGGCATAAGGC
Klf5	GTAACCCGGATCTGGAGAAG	CAGGTGCACTTGTAGGGCTT
Mcl-1	GCGTGTTATGCTCCCAGTTCC	TGCCAATCCAAGAATGCCAATCC
MS16	CTTGGAGGCTTCATCCACAT	ATATTCGGGTCCGTGTGAAG
PU.1	CCTTCGTGGGCAGCGATGGA	TGTAGCTGCGGGGGCTGCAC
Runx1	CACCGTCATGGCAGGCAAC	GGTGATGGTCAGAGTGAAGC
Tal1	AACAACAACCGGGTGAAGAG	CATTCACATTCTGCTGCCTC
**Genomic DNA**		
*Ikkb*	TCTGCGGTGGTCATAGGTCT	TCCTCTAGAAGCCTCCAGGAC
*RelA*	GCCGTGATGGATCTAGGGTC	TCCCCATTCAGTTCCCAAGC

Protein samples were subjected to Western blotting as described, [35] using the following antibodies IKKβ (sc34673), p65 (sc372), IκBα (sc371) (Santa Cruz Biotechnologies, Santa Cruz, CA), phospho-IκBα (2859) (Cell Signaling), β-catenin (610154) (BD Bioscience, San Jose, CA), and β-actin (AC15) (Sigma-Aldrich). Densitometric analysis of band intensity was perormed using ImageJ (National Institutes of Health Bethesda, MD USA).

### Statistical analysis

Quantitative data is presented as mean ± SEM from at least three independent repetitions. Statistical comparisons between groups were carried out using 2-tailed Student *t*-test. p values of < 0.05 were considered significant.

## Results

### IKKβ^Δ/Δ^ results in neutrophilia and decreased monocytes in mouse bone marrow

**Fig 1:**
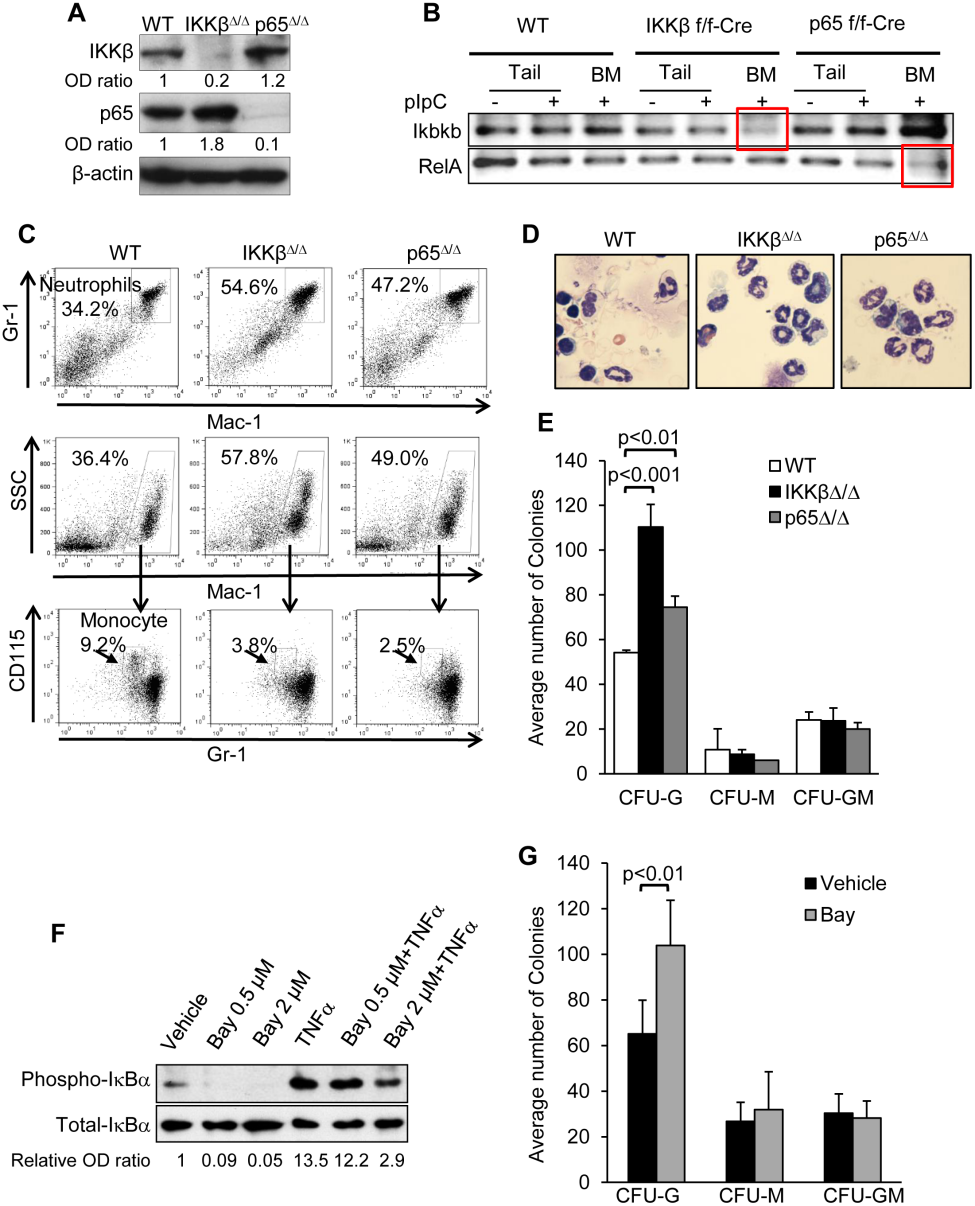
Loss of IKKβ results in increase granulocytes *in vivo* and *ex vivo*. **A**) Equal number of Lin^-^ bone marrow cells from wild-type (WT), IKKβ^Δ/Δ^, or p65^Δ/Δ^ mice were subjected to immunoblotting with the indicated antibodies. Representative gels with relative band intensity values are shown. **B**) Genomic DNA was extracted from the tail or bone marrow of mice with the indicated genotypes before or after pIpC injection and subjected to PCR analysis using primer sets that anneal to the excised fragment. **C**) Marrow cells from mice of the indicated genotypes were stained for Mac-1 and Gr-1 (top) and analyzed by FACS. Mac-1^+^ cells (middle) were stained for Gr-1 and CD115 (bottom). **D**) Peripheral blood leukocytesfrom 26 weeks old mice were subjected to Wright-Giemsa staining and representative morphology is shown. **E**) Equivalent numbers of mononuclear marrow cells from wild type (WT), IKKβ^Δ/Δ^, or p65^Δ/Δ^ mice were plated in methylcellulose with IL-3, IL-6, and SCF, and CFU-G, CFU-M, and CFU-GM colonies were enumerated 8 days later. The average number of colonies from 4 experiments is shown. **F**) Bone marrow cells were cultured with the indicated combinations of 20 ng/mL murine TNFα for 30 min, with or without 4 hrs pre-incubation with Bay 65–1942 at the indicted dose. Cell lysates were subjected to Western blotting with the indicated antibodies and the numbers below the blots indicate the relative band densities. **G**) Bone marrow cells were plated in methylcellulose with IL-3, IL-6, and SCF in the presence or absence of 0.5 μM Bay 65–1942 and colonies were enumerated 8 days later. The average number of colonies from 3 experiments is shown (right).

Injection of IKKβ(f/f);Mx1-Cre or p65(f/f);Mx1-Cre mice with poly(I:C) resulted in efficient reduction of these proteins in Lin^-^ marrow cells (Fig 1A). Using primers designed to anneal at the excised region (Table 1) efficient deletion of the *Ikbkb* or *RelA* genes could be demonstrated at the genomic DNA level (Fig 1B). The residual amplification may reflect presence of genomic DNA from Mx1-Cre non-expressing cells such as stromal cells. As previously reported [22,25,28], deletion of IKKβ was associated with myeloid expansion, markedly increased circulating neutrophils with splenomegaly, mild thrombocytosis, and mild anemia (Table 2). In contrast, p65^Δ/Δ^ mice have only modest splenomegaly and modest elevation of neutrophil counts that did not reach statistical significance, compared to wild type mice (Table 2). Neutrophilia was observed in the marrow of IKKβ^Δ/Δ^ and p65^Δ/Δ^ mice and these mice each also had decreased marrow monocytes (Fig 1C). Importantly, despite myeloid expansion IKKβ^Δ/Δ^ mice did not develop myeloid malignancy by 26 wks of age. Review of peripheral blood smears revealed marked neutrophilia but no circulating blast forms (Fig 1D) or excess of CD117^+^Mac1^+^Gr1^+^ cells (0.18±0.02%, 0.37±0.2%, 0.32±0.02%, p = NS for wild type, IKKβ^Δ/Δ^, or p65^Δ/Δ^ mice, respectively).

**Table 2:** Complete blood counts and spleen weights 6 wks after poly(I:C) injections.

	WT	IKKβ Δ/Δ	p65 Δ/Δ
**n**	10	10	10
**WBC (K/μL)**	16.0 ± 1.3	30.8 ± 5.7 *	18.0 ± 1.75
**NE (K/μL)**	4.1 ± 0.4	16.7 ± 4.1 **	5.5 ± 0.7
**RBC (M/μL)**	9.3 ± 0.3	7.4 ± 0.2 **	8.9 ± 0.24
**Hb (g/dL)**	12.7 ± 0.5	9.9 ± 0.3 **	12.8 ± 0.4
**PLT (K/μL)**	431.8 ± 49.9	649.2 ± 72 *	407.5 ± 38.3
**Spleen weight (g)**	0.093 ± 0.01	0.332 ± 0.07 **	0.11 ± 0.01 *

WBC denotes white blood cell count, NE neutrophils, RBC red blood cells, Hb hemoglobin, and PLT platelets.

* p ≤ 0.02,

** p ≤ 0.001.

Marrow cells from wild type, IKKβ^Δ/Δ^, or p65^Δ/Δ^ mice were plated in methylcellulose medium with IL-3, IL-6, and SCF to allow enumeration of myeloid progenitors. IKKβ deletion increased the number of CFU-G 2-fold, while deletion of p65 had only a modest effect (Fig 1E). Bay 65–1942, an inhibitor of IKKβ [36] markedly reduced IκBα phosphorylation (Fig 1F). Reproducibly, plating equal number of wild type bone marrow cells in methylcellulose medium with IL-3, IL-6, and SCF in the presence of Bay 65–1942 resulted in a significantly increased number CFU-G (Fig 1G), consistent with the effect of IKKβ gene deletion. Interestingly plating IKKβ^Δ/Δ^ marrow in the presence of Bay 65–1942 resulted in diminished number of CFU-G colonies (115 ± 14 vs. 80 ± 3.8, p = 0.02) presumably due to an enhanced off-target effect in the absence of IKKβ.

### IKKβ^Δ/Δ^ results in accumulation of myeloid progenitors and stem cells

**Fig 2:**
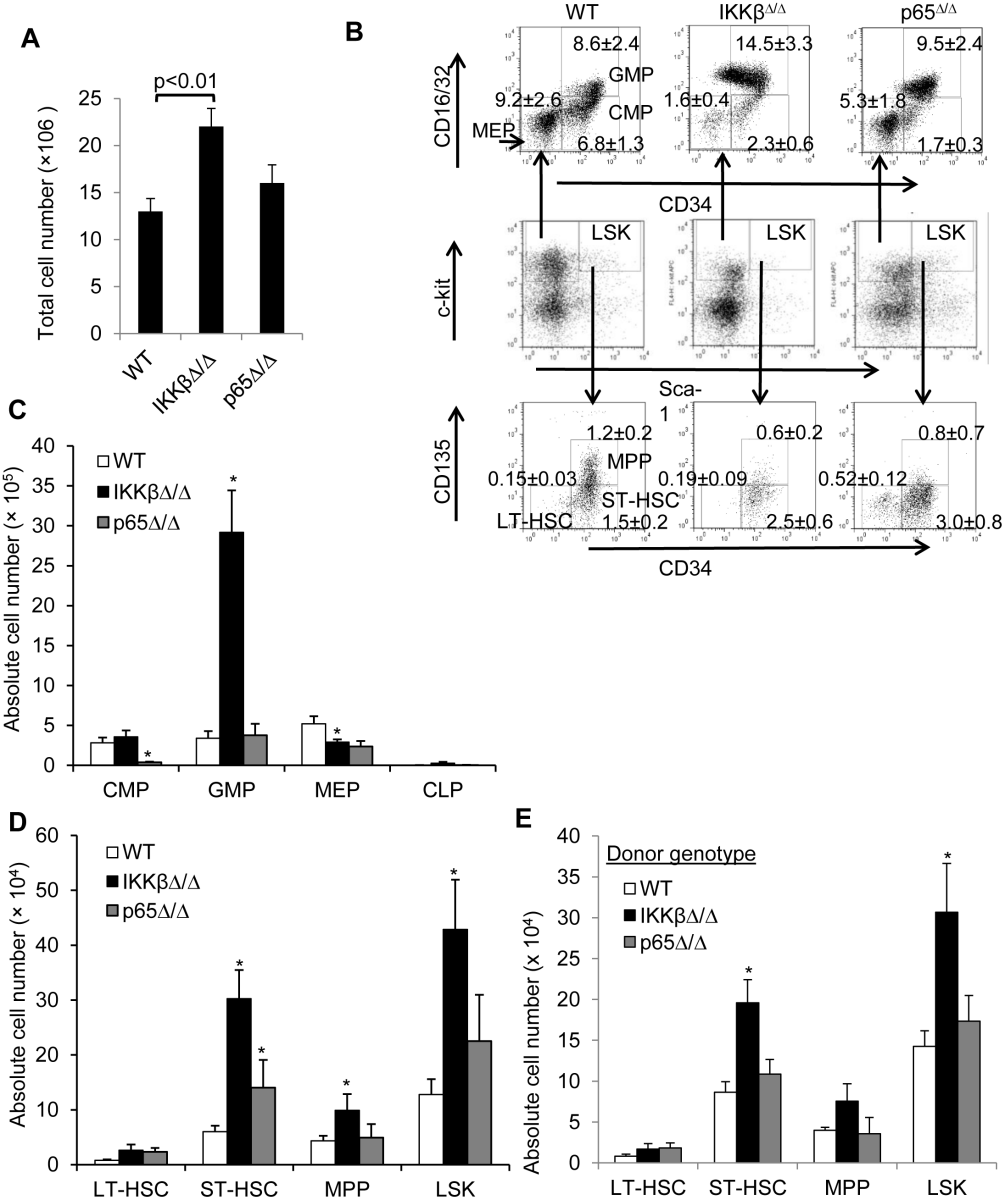
Deletion of IKKβ is associated with increased number of myeloid progenitors and hematopoietic stem cells. **A**) Bone marrow cells were harvested from the tibias of wild type or IKKβ^Δ/Δ^ mice, and the average number of cells is presented (n = 4). **B**) Bone marrow cells from wild-type (WT), IKKβ^Δ/Δ^ or p65^Δ/Δ^ mice were stained for lineage markers, Sca-1, c-Kit, CD34, CD16/32 and CD135. The indicated progenitor populations were identified and representative plots and the percent ± SEM of each population relative to total Lin^-^ marrow cells are shown (n = 4). **C**) The actual number of cells for the indicated progenitor populations per hind leg was calculated and averages from at least 4 experiments are shown. **D**) The average absolute number of multipotential progenitors (MPP) and long and short term hematopoietic stem cells (LT- and ST-HSC) per hind leg are shown (n = 4). **E**) CD45.1 mice were lethally irradiated and then intravenously injected with 1E6 CD45.2 marrow cells from wild type, IKKβ(f/f);Mx1-Cre, or p65(f/f);Mx-1-Cre mice. Eight wks after transplant mice were intraperitoneally injected with poly(I:C) for 7 doses starting four weeks after transplantation. Marrow harvested 6 week later and the average absolute number of the indicated progenitor and stem cell populations per hind leg are shown (n = 3).

We next utilized FACS to further compare the frequency of various hematopoietic progenitor populations in marrow cells from wild type, IKKβ^Δ/Δ^, and p65^Δ/Δ^ mice, including the LSK, common myeloid progenitor (CMP), granulocyte/monocyte progenitor (GMP), megakaryocyte/erythroid progenitor (MEP), and common lymphoid progenitor (CLP). Deletion of IKKβ using Mx1-Cre leads to a 2-fold increase in the total number of cells retrieved from the marrow (Fig 2A), in contrast to p65 deleted mice. Therefore, both the proportion of each subpopulation and the absolute number of cells in each fraction per hind leg is presented. As expected from their neutrophilia and as previously reported [25], IKKβ^Δ/Δ^ mice had significantly increased proportion and total number of GMPs. However, the absolute number of CMPs was similar and their proportion reduced (Fig 2B and 2C). As previously noted, loss of p65 is associated with a significantly reduced number of CMPs (Fig 2C) [26]. In addition, comparing wild type to mice lacking IKKβ or p65 we noted a significant decrease in the number and percentage of MEPs but no effect on the number of CLPs (1940 ± 490, 2540 ± 1700, or 3750 ± 1690 per hind leg, respectively) (Fig 2C). Notably, total numbers of multipotent progenitors (MPP) and the ST-HSC subpopulations of the LSK fraction are significantly increased in IKKβ^Δ/Δ^ mice (Fig 2B and 2D). The trend towards increased number of LT-HSC did not reach statistical significance (p = 0.1). To confirm a cell autonomous expansion of myeloid progenitors we harvested marrow from CD45.2 wild type, IKKβ(f/f);Mx1-Cre, or p65(f/f);Mx1-Cre mice and injected these into CD45.1, lethally irradiated congenic mice. Mice were exposed to poly(I:C) only after engraftment. Analysis of myeloid progenitors 6 weeks later revealed a significant 2-fold expansion of the LSK and ST_HSC fractions in the absence of IKKβ but not p65, and in addition revealed an increased numbers of MPPs or LT-HSC approached statistical significance in the absence of IKKβ (p = 0.1) (Fig 2E). Of note, mice transplanted with p65^Δ/Δ^ marrow do not reconstitute hematopoiesis and die.

### IKKβ deletion is associated with reduced apoptosis and altered cell cycle distribution in progenitor populations

**Fig 3:**
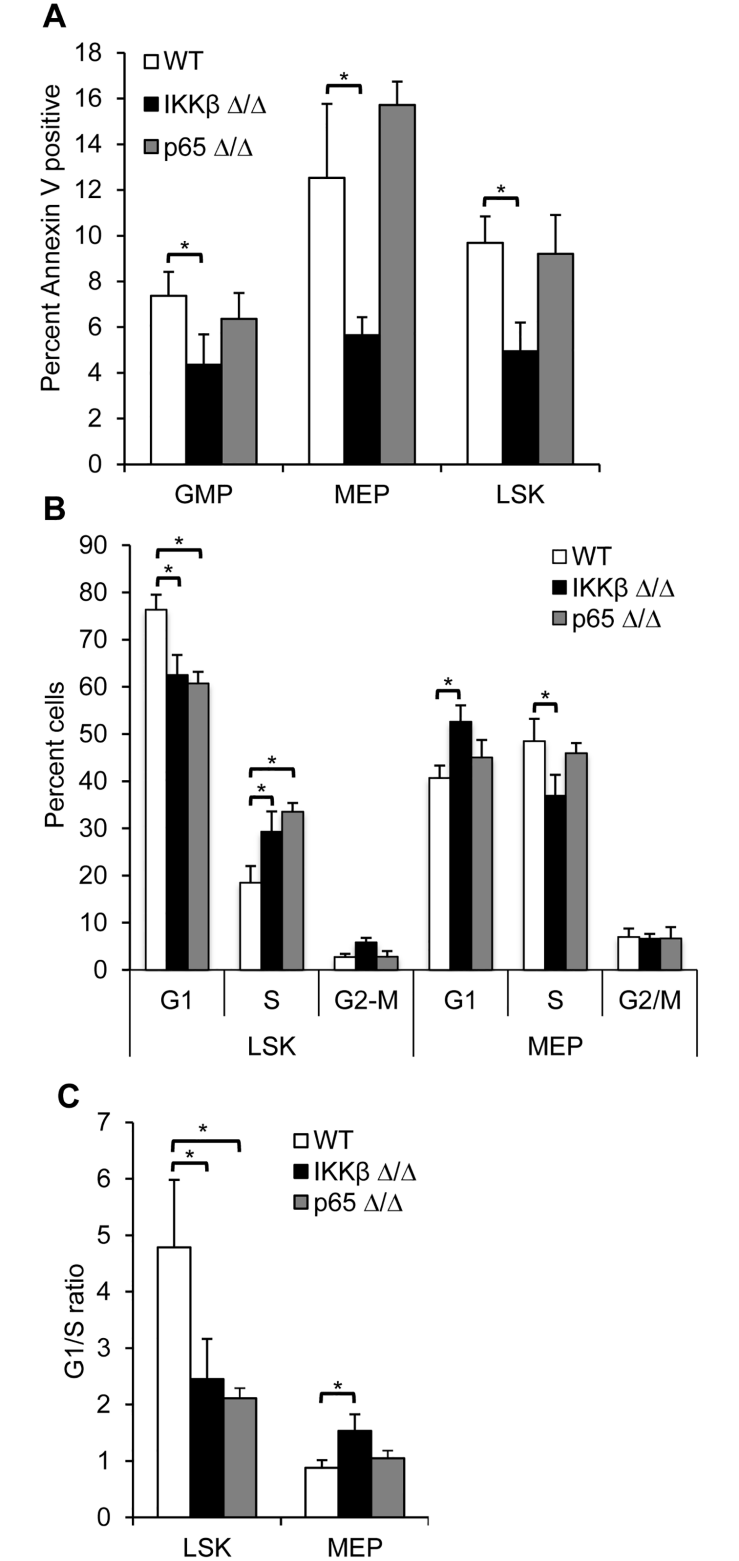
IKKβ deletion is associated with reduced apoptosis and altered cell cycle distribution. **A**) Bone marrow cells from wild type, IKKβ^Δ/Δ^, or p65^Δ/Δ^ mice were stained for lineage markers, Sca-1, c-Kit, CD34, CD16/32, and Annexin V. The average proportion of cells positive for Annexin V for each of the indicated progenitor populations is shown (n = 3). **B**) The indicated populations from wild type, IKKβ^Δ/Δ^, or p65^Δ/Δ^ mice were sorted, stained with propidium iodide and DNA content was analyzed by flow cytometry. The mean distribution +/- SEM of cells in G1, S, or G2/M from four experiments are presented as well as **C**) the ratio of the proportion of cells in G1 over S phase from four experiments are presented (n = 4). *-denotes p<0.01.

Change in survival pathways could contribute to the alteration seen in hematopoietic progenitor populations. Indeed the proportion of Annexin-V positive GMP, MEP, or LSK cells is significantly lower in marrow cells from IKKβ^Δ/Δ^ compared to wild type mice, while the rate of apoptosis in p65^Δ/Δ^ progenitors is similar to wild type cells (Fig 3A). However, this does not explain the observed altered progenitor ratios, as the proportion of apoptotic IKKβ^Δ/Δ^ MEPs is lower despite their reduced number in those mice. Enhanced proliferation was demonstrated in IKKβ^Δ/Δ^ GMPs [28], therefore, we investigated the effect IKK deletion on LSK and MEP cell cycle distribution (Fig 3B). IKKβ or p65 deficient LSK cells were found to have an increased proportion of cells in S phase and fewer in G1, compared to wild type cells. In contrast, a lower proportion of IKKβ^Δ/Δ^ MEPs were in S phase and an increased percent in G1 compared to wild type or p65^Δ/Δ^ MEPs. We also plotted these data as a G1/S ratio (Fig 3C). These data suggest that reduced rate of G1 to S progression contributes to HSC expansion and to diminished MEPs in IKKβ deleted marrow cells.

### IKKβ^Δ/Δ^ mice have an erythroid defect

**Fig 4:**
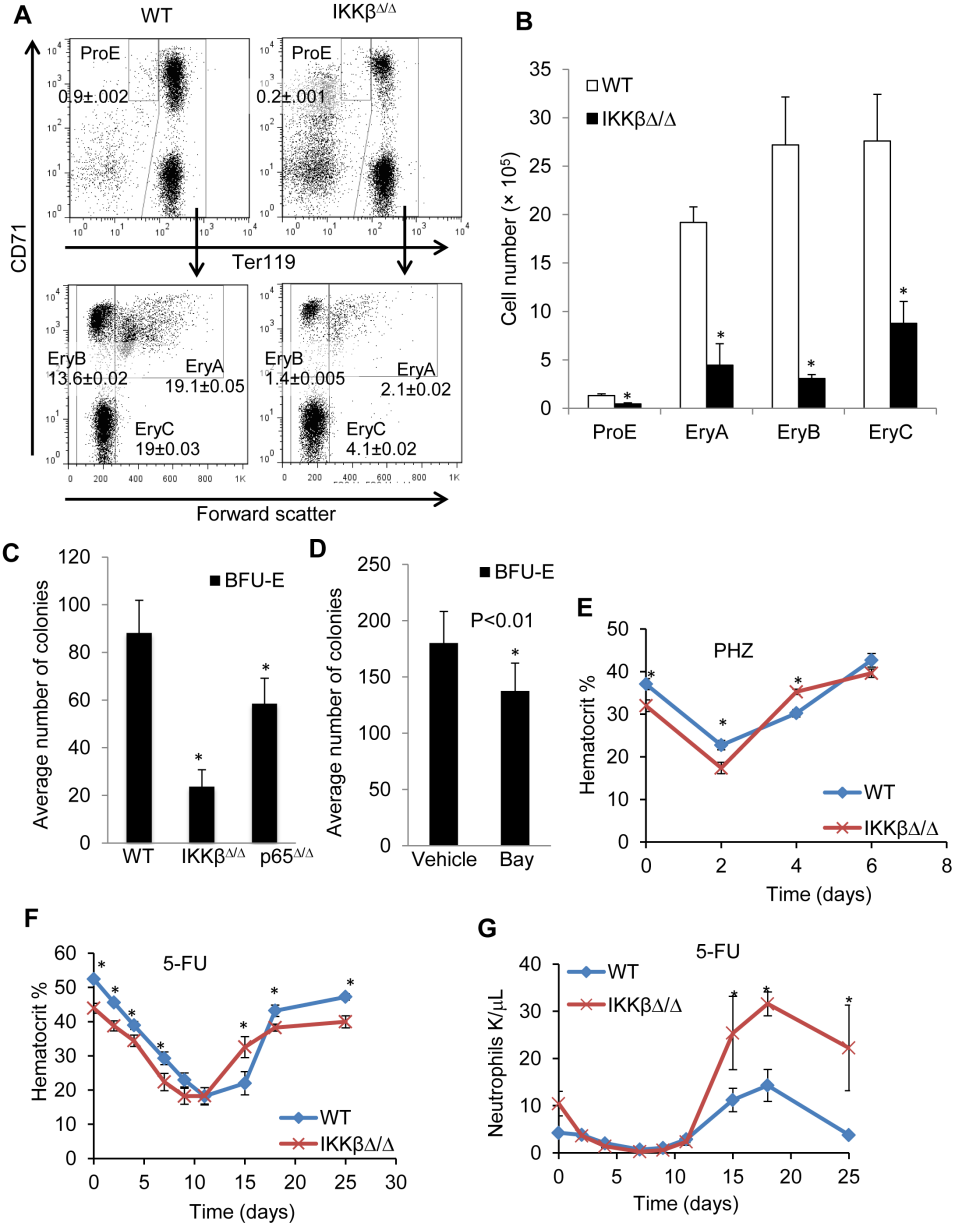
IKKβ deletion is associated with impaired erythropoiesis. **A**) Bone marrow cells from wild-type (WT) or IKKβ^Δ/Δ^ mice were stained for CD11b, CD45, Ter119 and CD71. CD11b^-^;CD45^-^ cells were gated and the proportion of proerythroblasts, basophilic, polychromatic, and orthochromatic erythroblasts in nucleated bone marrow cells is shown on representative FACS plots. **B**) The average number of cells of each of erythroid subpopulation per hind leg is shown (n = 4). **C**) Equal number of marrow cells from wild type, IKKβ^Δ/Δ^ or p65^Δ/Δ^ mice were plated in methylcellulose under conditions promoting erythroid maturation, and BFU-Es were enumerated after 8 days. Average number of colonies from 3 experiments is shown. **D**) Wild type bone marrow cells were plated in methylcellulose in the presence or absence of 1 μM Bay 65–1942, and BFU-Es were enumerated 8 days later (n = 3). Hematocrit was assessed in wild type or IKKβ^Δ/Δ^ mice after induction of hemolysis with **E**) PHZ 200 mg/kg or F) challenge with 250 mg/kg 5-FU. **G**) Neutrophil recovery after 5-FU. *-denotes p<0.01. **denotes p<0.001.

In addition to decreased total MEPs (Fig 2C), targeted deletion of IKKβ results in mildly decreased hemoglobin (Table 2) and hematocrit, which was 43.9 ± 0.6 vs. 52.5 ± 0.6 (n = 9, p<0.001) for IKKβ^Δ/Δ^ vs. wild type mice. Erythroid precursor subsets in the marrow can be defined based on Ter119, CD71 surface expression and forward scatter [37] as proerythroblasts (ProE, Ter119^med^CD71^high^FSC^high^), basophilic (EryA, Ter119^high^CD71^high^FSC^high^), late basophilic and polychromatic (EryB, Ter119^high^CD71^high^FSC^low^), or orthochromatic erythroblasts (EryC, Ter119^high^CD71^low^FSC^low^). We observed significantly decreased total number of each of these subsets in marrow from IKKβ^Δ/Δ^ mice (Fig 4A and 4B). We next plated equal number of marrow cells from wild type or IKKβ^Δ/Δ^ mice in methylcellulose with erythropoietin and enumerated BFU-E after 8 days. On average, IKKβ^Δ/Δ^ had approximately 4-fold lower number of BFU-E (Fig 4C), although colony sizes were similar between the two genotypes (not shown). Wild type marrow cells were also evaluated for BFU-E formation in the presence 1 μM of the IKKβ inhibitor Bay 65–1942 or vehicle control. Enumeration of colonies after 8 days revealed that IKKβ inhibition in wild type marrow again significantly reduced the number of BFU-E (Fig 4D). To further define the potential functional consequence of the decreased erythroid progenitors in the absence of IKKβ we challenged wild type and IKKβ^Δ/Δ^ mice with 5-fluorouracil (5-FU) (Fig 4E) or phenylhydrazine (PHZ) (Fig 4F) and followed hematocrit recovery with serial blood counts. The rate of hematocrit decline was similar, and IKKβ^Δ/Δ^ mice showed a robust recovery with significantly higher average hematocrit than wild type mice on day 4 after PHZ and on day 15 after 5-fluorouracil exposure (Fig 4E and 4F). Ultimately the hematocrit of IKKβ^Δ/Δ^ mice returned to a mildly decreased baseline (Fig 4E and 4F). We also examined the recovery of neutrophils after 5-fluorouracil exposure—serial measurement of neutrophil counts demonstrated a similar recovery time in wild type or IKKβ^Δ/Δ^ mice (Fig 4G).

### Deletion of IKKβ is associated with long term repopulation advantage

**Fig 5:**
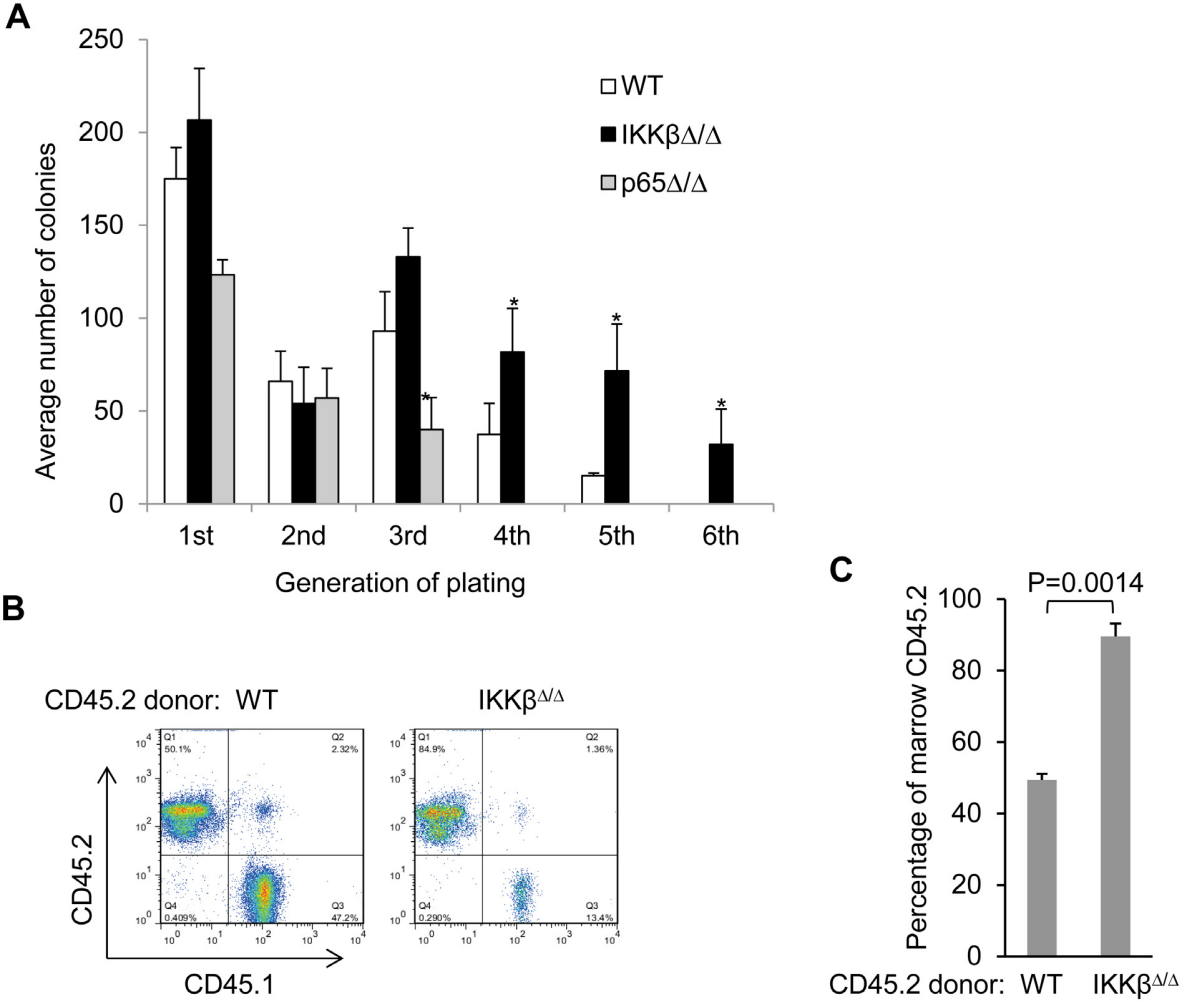
Deletion of IKKβ is associated with replating and repopulation advantage. **A**) Equal number of wild type, IKKβ^Δ/Δ^ or p65^Δ/Δ^ bone marrow cells were plated and myeloid CFUs were enumerated and replated every 7 days. The average number of colonies at the indicated replating generation is shown. **B, C**) Lethally irradiated CD45.1 mice were injected with 5E5 CD45.2 marrow cells from wild type or IKKβ^Δ/Δ^ along with 5E5 CD45.1 marrow cells. Engraftment of bone marrow was assessed using FACS analysis for CD45.1 and CD45.2. Representative marrow FACS plots and the average proportion of CD45.2 engraftment in the marrow 20 weeks after transplant is shown (n = 5).

We noted a significant increase in the number of LSK and ST-HSC in IKKβ^Δ/Δ^ mice and a trend towards increased FACS-defined LT-HSC. We therefore investigated whether these increases are also reflected in functional assays. In the serial replating assay, IKKβ^Δ/Δ^ myeloid CFUs were efficiently replated for 6 rounds while wild type marrow could not be replated efficiently more than 4 times (Fig 5A). Interestingly, p65^Δ/Δ^ marrow progenitors have a diminished replating potential and could not be replated beyond the third round (Fig 5A). Serial replating of wild type marrow in the presence of the IKKβ inhibitor Bay 65–1942 could not be extended beyond four generations. This difference from marrow obtained from the IKKβ^Δ/Δ^ mice may be explained by off target effects of the chemical inhibitor or due to the fact that *in vivo* expansion of HSC could not be replicated by a short exposure to the inhibitor *in vitro*.

A competitive repopulation experiment was carried to test the repopulation potential of HSC lacking IKKβ. Ours and previous experience demonstrate that HSCs lacking p65 do not reconstitute transplant recipients in a competitive situation [26]. We therefore studied only IKKβ^Δ/Δ^ donor mice. Lethally irradiated congenic CD45.1 mice were transplanted with equal numbers of unsorted CD45.2 IKKβ^Δ/Δ^ marrow and CD45.1 competitor wild type marrow cells. Relative engraftment was assessed using flow cytometry on marrow 20 wks after transplantation a time point that reflects LT-HSC contribution [38,39]. Cells lacking IKKβ had a marked advantage and represented 89.6 ± 3.6% of marrow cells at 20 wks post-transplantation (Fig 5B and 5C).

### Deletion of IKKβ is associated with altered expression of hematopoietic transcription factors

**Fig 6:**
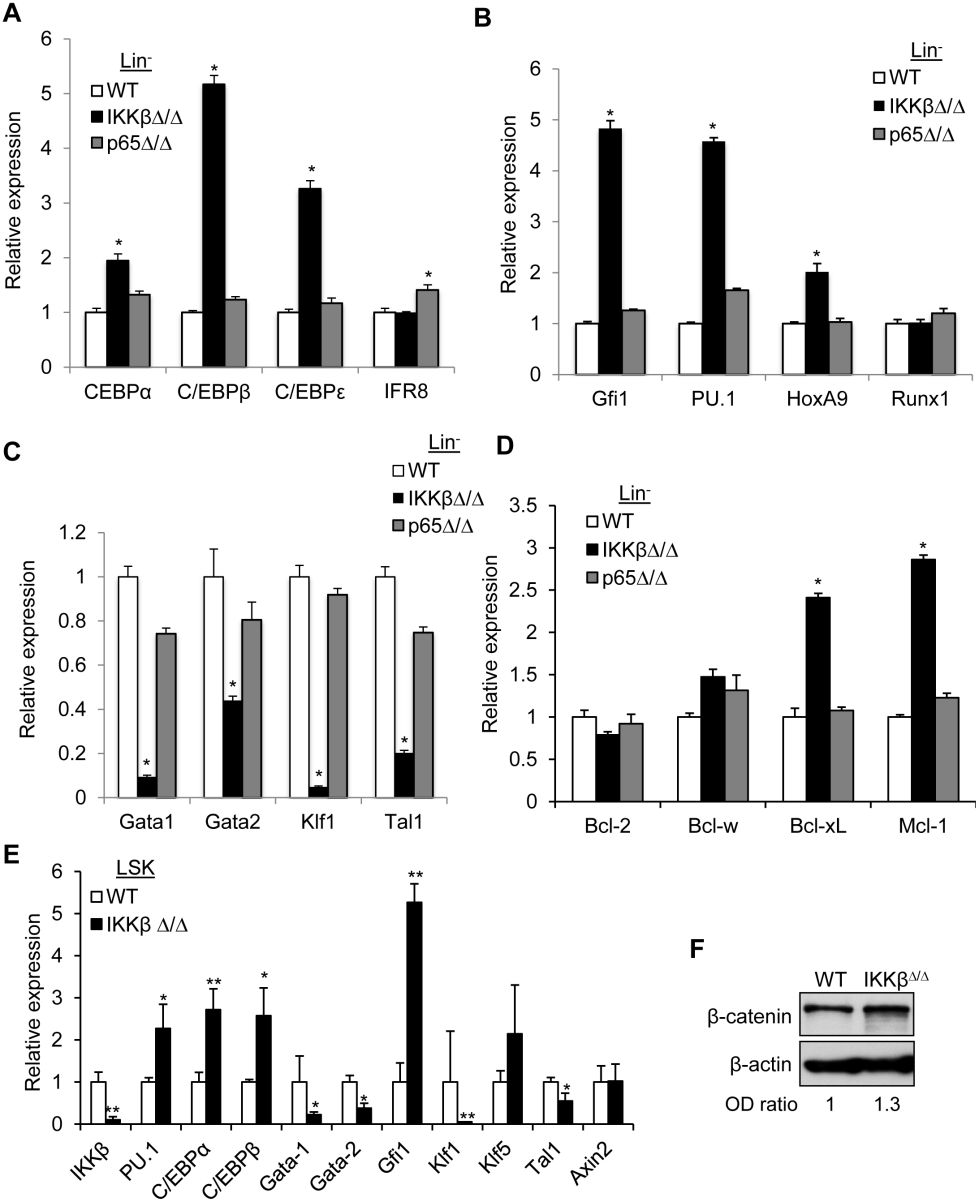
Deletion of IKKβ is associated with altered expression of hematopoietic transcription factors. Total cellular RNA was isolated from Lin^-^ marrow cells of mice with the indicated genotypes and the expression of **A**) myeloid differentiation associated genes, **B**) hematopoietic transcription regulators **C**) transcription factors regulating erythropoiesis, and **D**) anti-apoptotic Bcl2 family members was analyzed using qRT-PCR. Average relative expression from 3–4 independent experiments is shown. **E**) LSK cells were isolated by FACS from wild type or IKKβ^Δ/Δ^ mice, RNA was extracted and subjected to qRT-PCR. *-denote p<0.01. **denotes p<0.001. **F**) Cell lysates were obtained from wild-type (WT) or IKKβ^Δ/Δ^ Lin^-^ bone marrow cells and subjected to Western blot analysis with the indicated antibodies. Representative gels with relative band intensity values are shown.

Gene expression of key transcription factors was analyzed in Lin^-^ marrow cells from wild type, IKKβ^Δ/Δ^, or p65^Δ/Δ^ mice. In accordance with the significant neutrophilia in IKKβ^Δ/Δ^ mice, members of the C/EBP transcription factor family were highly expressed in IKKβ^Δ/Δ^ but not p65^Δ/Δ^ Lin^-^ marrow cells (Fig 6A). Gfi1, PU.1, and HoxA9 are expressed in immature myeloid cells and were also increased in Lin^-^ cells from IKKβ^Δ/Δ^ mice, whereas Runx1 was unchanged (Fig 6B). In addition, IKKβ^Δ/Δ^ cells have significantly lower expression of key regulators of erythropoiesis, including Gata1, Gata2, Klf1 and Tal1 (Fig 6C). In contrast, expression of mRNAs encoding these myeloid or erythroid transcription factors genes is similar in wild type and p65^Δ/Δ^ mice.

Since we observed reduced apoptosis in IKKβ^Δ/Δ^ progenitors, we also examined the levels of several pro-survival Bcl2 family members that are known NF-κB transcriptional targets. Mcl1 and Bcl-xL transcript levels were increased 3-fold in IKKβ^Δ/Δ^ cells but the expression of Bcl2 or Bcl-w anti-apoptotic family member was unaffected by IKKβ or p65 deletion (Fig 6D).

We also extracted RNA from sorted wild type or IKKβ^Δ/Δ^ LSK cells and again analyzed expression of key transcription factors. Notably, similar to the Lin^-^ population, PU.1, C/EBPα, and Gfi1 were significantly increased in IKKβ^Δ/Δ^ LSKs whereas Gata1, Gata2, Klf1, and Tal1 were significantly diminished (Fig 6E). These data suggest that altered expression of transcription factors occurring in the LSK population, prior to progression to GMP or MEP, is responsible for the observed skewing of differentiation upon IKKβ gene deletion.

As IKKβ phosphorylates β-catenin resulting in its ubiquitin dependent proteasomal degradation [7], we explored the activity of the canonical Wnt pathway. Axin 2 expression was similar in wild type and IKKβ^Δ/Δ^ LSKs (Fig 6E), as was the level of β-catenin protein in wild type and IKKβ deficient Lin- cells (Fig 6F). These data suggest that differential activation of the canonical Wnt pathway is not responsible to the phenotype of IKKβ^Δ/Δ^ mice.

## Discussion

The current study confirms myeloid expansion in the absence of IKKβ and reveals also intrinsic increased myeloid progenitors proliferative capacity, impaired erythropoiesis associated with reduced MEPs G1 to S phase progression, and functional LT-HSC expansion upon IKKβ deletion in adult mice.

IKKβ activates of the canonical NF-κB system via phosphorylation of IκBs. In addition, IKKβ directly phosphorylates p65 to enhance its transcriptional activity and modifies co-repressors such as SMRT to de-repress NF-κB target genes [40–42]. Therefore, a similarity might be expected between IKKβ^Δ/Δ^ and p65^Δ/Δ^ hematopoiesis. Indeed, these mice share neutrophilia, myeloid expansion, and expansion of MPP and ST-HSCs. Interestingly, however, we also noted important differences including an opposing functional effect on LT-HSCs and skewing of differentiation, with myeloid over erythroid commitment and an associated pattern of transcription factor gene expression even in LSK cells unique to IKKβ^Δ/Δ^ mice. In direct comparison using Mx1-Cre deletion, IKKβ^Δ/Δ^ mice have a nearly two fold increase in the absolute number of MPP and ST-HSC cells compared to p65^Δ/Δ^. In addition to quantitative expansion based on FACS analysis, functionally, IKKβ^Δ/Δ^ myeloid progenitors display greater replating capacity and their LT-HSC have a repopulation advantage in contrast to myeloid CFUs or LT-HSCs lacking p65. Major differences between IKKβ or p65 deficient hematopoiesis are summarized in Table 3. Future efforts will be devoted to identifying NF-κB-independent pathways that mediate the hematopoietic effect of IKKβ deletion.

**Table 3:** Effects of IKKβ or p65 deletion on hematopoiesis.

**Peripheral blood counts**	**IKKβ^Δ/Δ^**	**p65^Δ/Δ^**
neutrophils	**↑↑↑**	**↑**
hemoglobin	**↓**	—
**Spleen size**	**↑↑**	**↑**
**Marrow progenitors**	**↑↑** GMP, **↓** MEP Enhanced re-plating	Similar to WT Diminished re-plating
**HSC**		
number	**↑** LT-HSC, **↑** ST-HSC	**↑** LT-HSC, **↑** ST-HSC
function	Repopulation advantage	Repopulation disadvantage26

It was previously shown that the marrow from IKKβ^Δ/Δ^ mice results in neutrophilia when transplanted into wild type recipients [28]. We now further demonstrate a cell autonomous expansion of LSK and phenotypic ST-HSCs in this setting. An advantage of IKKβ^Δ/Δ^ over wild type marrow in serial transplantation in future experiments would further support our conclusion that absence of IKKb enables LT-HSC expansion. By deleting the *Ikbkb* gene after recovery from transplant we minimized a potential contribution of differential engraftment or of pre-transplant deletion of IKKβ in marrow stroma to the observed stem cell phenotypes. Moreover, since marrow cells lacking p65 cannot be transplanted effectively [26], this approach allowed the evaluation of the cell autonomous development of p65^Δ/Δ^ marrow.

IKKβ^Δ/Δ^ mice have marked myeloid expansion manifesting as neutrophilia, splenomegaly, and increased total numbers of marrow cells. In addition, we now show that IKKβ deficient myeloid progenitors have skewed lineage commitment to GMP over MEPs. Both the proportion and the total number of GMPs is higher in IKKβ^Δ/Δ^ mice and the reverse is true for MEPs. MEPs from IKKβ^Δ/Δ^ mice have diminished G1 to S phase cell cycle progression which may contribute to their lower number. Despite this myeloid skewing, IKKβ^Δ/Δ^ mice recovered from anemia or neutropenia induced by either 5-FU or PHZ exposure with kinetics similar to wild type mice, indicating that IKKβ deletion does not impair stress erythropoiesis or myelopoiesis.

The determination of myeloid versus erythroid commitment is governed by transcription factors which serve as master regulators. Gata1 or PU.1 direct HSCs towards erythroid or myeloid/lymphoid differentiation, respectively [43,44] through the activation of downstream lineage genes and via their reciprocal suppression [45–47]. C/EBPα then further directs myeloid commitment [48]. Indeed we find a significantly decreased Gata1 and increased PU.1 and C/EBPα levels in IKKβ^Δ/Δ^ Lin^-^ cells. Importantly, a similar expression pattern is also observed in LSK cells, suggesting an early skewing of development. In contrast to the myeloid expansion and neutrophilia associated with loss of IKKβ or p65, we have previously shown that loss of NF-κB p50 results in impaired granulopoiesis due to reduced expression of C/EBPα [49].

The neutrophilia in IKKβ^Δ/Δ^ mice is driven by strong stimulation by cytokines, including G-CSF, inducing a marrow environment analogous to stress granulopoiesis [25,28] which is regulated by C/EBPβ [50]. Notably, C/EBPβ is increased more than C/EBPα upon IKKβ-gene deletion in Lin^-^ marrow cells. Erythroid differentiation depends on Gata1 binding its target genes at promoters occupied by Klf1 or SCL/Tal1 [51–53]. Our data demonstrate reduction of each of these regulators of erythropoiesis in Lin^-^ cells lacking IKKβ, and reduction of Gata1, Gata2, Klf2, and Tal1 in LSK cells.

Surprisingly, p65 deletion was not associated with increased apoptosis. Even more striking, we observed significantly less apoptosis in IKKβ deficient LSK cells compared to wild type that may be in part responsible for the expansion of HSC in IKKβ^Δ/Δ^ mice. Although inhibition of IKKβ in myeloid progenitors increases their susceptibility to TNFα induced apoptosis *in vitro* [25], our findings are consistent with previously observed prolonged survival of IKKβ^Δ/Δ^ neutrophils [28]. Protection from apoptosis is explained in part by the increased levels of Mcl1, whose expression in hematopoietic stem cells is critical [54,55], and increased Bcl-xL. IKKβ may partly regulate apoptosis in an NF-κB independent manner, for example, it mediates oxidative stress induced apoptosis through association with p85 S6K1, phosphorylation of Mdm2, and accumulation of p53 [56]. Absence of these pathways may explain the different antiapoptotic phenotype in IKKβ^Δ/Δ^ versus p65 deficient marrow cells and may contribute to their different HSC phenotypes, Interestingly, IKKβ-deletion is also associated with decreased apoptosis in MEPs suggesting that resistance to apoptosis plays only a limited role in IKKβ^Δ/Δ^- associated erythroid defect. In contrast, deletion of IKKβ or p65 has similar effect on G1 to S cell cycle progression of LSK cells, suggesting that the effect on the cell cycle may be contribute to the similar neutrophilia or myeloid expansion that is seen in these mice and that it likely plays a limited role in the specific HSC phenotype of IKKβ^Δ/Δ^ mice.

As noted earlier, IKK inhibitors are being evaluated for therapeutic use. Our data suggests that although development of such agents is primarily motivated by their potential blockade of cannonical NF-κB activation, some of the effects will be related to NF-κB-independent targets of IKK. Our findings suggest that effective, prolonged systemic inhibition of IKKβ may result in significant effects on the hematopoietic system, including change in levels of key transcription factors that regulate lineage commitment and skewing towards myeloid over erythroid differentiation. Of note, our findings also indicate that IKKβ inhibition is unlikely to be associated with injury to early hematopoietic stem and progenitor cells, and the normal recovery of neutrophil counts after a myelosupressive chemotherapy challenge of IKKβ^Δ/Δ^ mice suggests that combining IKKβ inhibition with chemotherapy will likely be tolerated without excessive myelotoxicity.
